# Short time guided bone regeneration using beta-tricalcium phosphate with and without fibronectin – An experimental study in rats

**DOI:** 10.4317/medoral.23564

**Published:** 2020-05-10

**Authors:** Mª Ángeles Sánchez-Garcés, Octavi Camps-Font, Jaume Escoda-Francolí, Fernando Muñoz-Guzón, Jorge Toledano-Serrabona, Cosme Gay-Escoda

**Affiliations:** 1 MD, DDS, MS, PhD, EBOS. Lecturer in Oral Surgery, Professor of the Master Degree Program in Oral Surgery and Implantology, Researcher of Institut d'Investigació Biomedica de Bellvitge (IDIBELL Institute), Faculty of Dentistry, University of Barcelona, Spain; 2 DDS, MS. Associate Professor of Oral Surgery, Professor of the Master Degree Program in Oral Surgery and Implantology, Researcher of Institut d'Investigació Biomedica de Bellvitge (IDIBELL Institute), Faculty of Dentistry, University of Barcelona, Spain; 3 DDS, MS, PhD. Master in Oral Surgery and Implantology; 4 DDS, MS, PhD. Associate Professor. Department of Veterinary Clinical Sciences, University of Santiago de Compostela, Spain; 5 DDS. Fellow of Master’s Degree of Oral Surgery and Implantology, Faculty of Dentistry, University of Barcelona, Spain; 6 MD, DDS, MS, PhD, EBOS, OMFS. Chairman and Professor of Oral and Maxillofacial Surgery, Faculty of Dentistry, University of Barcelona. Researcher and coordinator of the IDIBELL Institute; Director of the Master of Oral Surgery and Implantology (EFHRE International University /FUCSO); Director of the Dentistry, Oral and Maxillofacial Department of Centro Médico Teknon, Barcelona, Spain

## Abstract

**Background:**

The aim of this histomorphometric study was to assess the bone regeneration potential of beta-tricalcium phosphate with fibronectin (β-TCP-Fn) in critical-sized defects (CSDs) in rats calvarial, to know whether Fn improves the new bone formation in a short time scope.

**Material and Methods:**

CSDs were created in 30 Sprague Dawley rats, and divided into four groups (2 or 6 weeks of healing) and type of filling (β-TCP-Fn, β-TCP, empty control). Variables studied were augmented area (AA), gained tissue (GT), mineralized/non mineralized bone matrix (MBM/NMT) and bone substitute (BS).

**Results:**

60 samples at 2 and six weeks were evaluated. AA was higher for treatment groups comparing to controls (*p* < 0.001) and significant decrease in BS area in the β-TCP-Fn group from 2 to 6 weeks (*p* = 0.031). GT was higher in the β-TCP-Fn group than in the controls expressed in % (*p* = 0.028) and in mm2 (*p* = 0.011), specially at two weeks (*p*=0.056).

**Conclusions:**

Both β-TCP biomaterials are effective as compared with bone defects left empty in maintaining the volume. GT in defects regeneration filed with β-TCP-Fn are significantly better in short healing time when comparing with controls but not for β-TCP used alone in rats calvarial CSDs.

** Key words:**Bone regeneration, biomaterials, experimental design, histology.

## Introduction

The outcomes of dental implant treatment are high but compromised when there are alveolar bone defects. In these cases, regeneration procedures are frequently required before or at the time of implant placement.

Autogenous bone grafts are still considered as the gold standard due to their biological properties (1). However, the increased morbidity, the limited amount of tissue graft available from the donor site and a high resorption rate lead to use allogenic, xenogenic or alloplastic materials as alternatives (2-5).

Alloplastic material, such as, hydroxyapatite (HA) and beta-tricalcium phosphate (β-TCP) have been widely studied and can be used instead of bone grafts due to its excellent biocompatibility and osteoconductivity. Several experimental studies have evaluated the physical properties and bone regeneration (BR) effects of HA and β-TCP (6-9).

In recent years, extensive experimental research has focused to accelerate BR (10-13) and tissue engineering using combinations of cells, scaffolds and bioactive factors to treat skeletal defects (14-17).

Fibronectin (*Fn*) is a glycoprotein of the extracellular matrix that promotes cell adhesion and differentiation and has been used in combination with biomaterials for improving proliferation and differentiation of osteoblasts cultivated on composite scaffolds (16,17). Furthermore, it has been shown that anodized titanium implants treated with fibroblast growth factor-*Fn* also enhanced osseointegration (18).

A previous study has tested the bone regeneration potential of β-TCP-*Fn* with autologous adipose-derived stem cells (β- TCP-*Fn*-ADSCs) in CSDs of alveolar ridges in a dog model (19) and in dehiscence-type defects associated with dental implants (20). The use of ADSCs does not seem to improve the area of bone regeneration and bone-implant contact (BIC) and did not entail a significant advantage as compared with β-TCP-*Fn* alone.

In other study in rats, β-TCP-*Fn* was not significantly more effective than β-TCP alone for improving the volume of regenerated bone in CSDs at 6 and 8 weeks, although bone turnover was higher in the β-TCP-*Fn* and differences between the use of β-TCP-*Fn* or β-TCP were observed at 8 weeks (4.25 [1.39] mm2 and 2.38 [0.88] mm2 respectively; *P* = 0.004) in terms of gained tissue (mineralized bone matrix plus bone graft). No other significant differences between both grafting groups were found (21).

**Figure 1:**
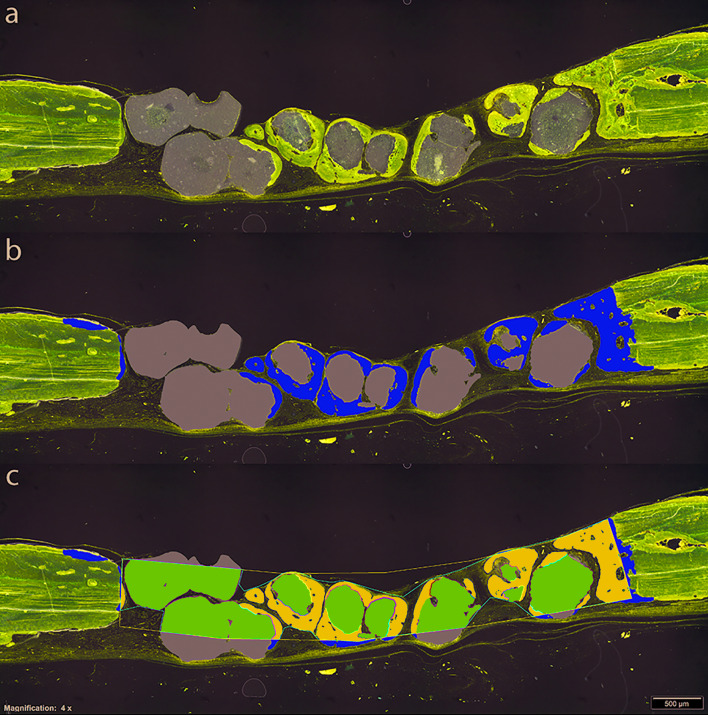
Histomorphometric analysis. Definition of the regions of interest (ROI) for the digital tissue differentiation procedure. Original image (a). Using a digital pen, the proportions occupied by new mineralized bone matrix (MBM, yellow) and bone substitute (BS, grey) were coloured. Defect area region (DA) was defined as the area occupied by the bone extracted during the surgery. Firstly, the interface between new and pristine bone was detected, outlined and finally linked following the curvature of the skull with straight lines (black and blue polygons). The increase in the cortical thickness caused by periosteal reaction was avoided (b). Augmented area region (AA) was outlined (red polygon) following the surface of the DA occupied by MBM and BS. The image analysis software calculated the area of MBM and BS within the AA. Percentage of graft in contact with new bone within the AA was manually determined using a digital pen (c). Magnification x40. Lévai-Laczkó staining.

The aim of this histomorphometric study was to assess BR potential of β-TCP with and without *Fn* (β-TCP-*Fn*) in a short time period (2 and 6 weeks) by comparing them with a control in calvarial CSDs in an experimental rat model, when all defects are covered by a native collagen barrier membrane (Fig. 1).

It was hypothesized that β-TCP-*Fn* would improve bone formation as compared with β-TCP alone at 2 weeks in this experimental rat model.

## Material and Methods

- Material

The coating process of Beta-tricalcium phosphate (β-TCP) 99% pure (KeraOs®, Keramat, A Coruña, Spain), was the same previously reported for the authors (19-21).

- Study design

The present reporting of *in vivo* animal experiment followed the ARRIVE guidelines (22). Animal procedures were approved by the Ethics Committee on Animal Research (CEEA 346-12) of the University of Barcelona, Barcelona (Spain). Thirty male ex-reproductive Sprague Dawley rats (250-300g, 14 weeks’ old) were included in a prospective controlled study.

- Surgical protocol

The surgical protocol of this experimental study was previously described in detail by Escoda-Francolí *et al*. (21). Briefly, after the animals were anesthetized a cranial cutaneous incision was done in an antero-posterior direction. When calvarial bone was exposed, two bicortical cirtical-sized defects were created using a trephine bur of 5 mm of external diameter in both parietals. Out of 30 defects, half of these were filled with material (β-TCP or β-TCP-*Fn*) and 30 were left empty as controls in the contralateral side. All defects were covered with a native bovine collagen membrane (Collagen-Klee®, Medical Biomaterials Products GmbH, Neustadt, Glewe, Germany), and the surgical field was closed by primary intent.

Finally, four study groups were obtained as follows: β-TCP-*Fn*/2 weeks, β-TCP/2 weeks, β-TCP-*Fn*/6 weeks, β-TCP/6 weeks.

- Histological preparation and histomorphometrical analysis

Histological samples of the skull portion surrounding the defect and the histomorphometric studies were performed by an experienced investigator (FM) who was blinded to the experiment were prepared following guidelines previously published as in previous published studies (19-21).

**Figure 2:**
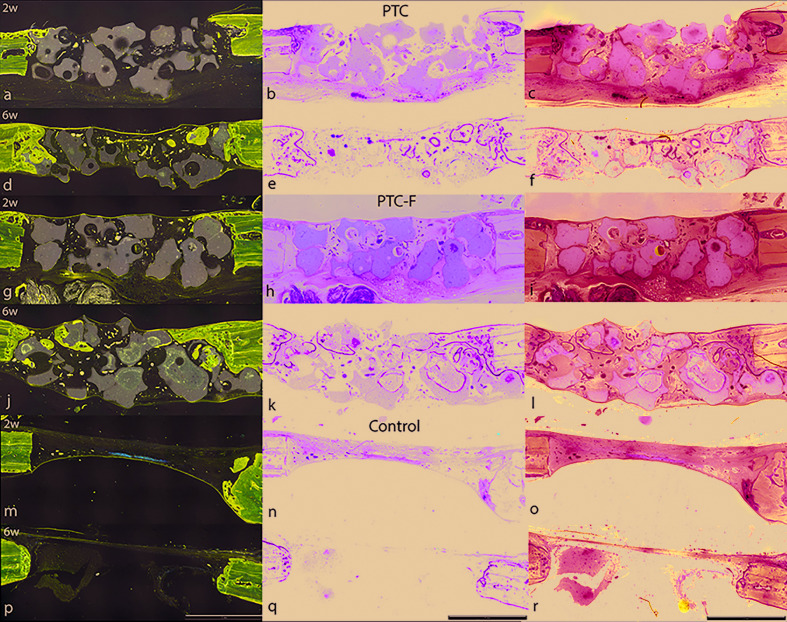
Representative images of the defects filled with TCP, TCP-*Fn* and control at 2 and 6 weeks. One week before sacrificing the animals, they received a dose of fluorochrome (tetracycline) subcutaneously (right side images obtained with different wavelength filters). It can be observed differences between the control and the treated sites concerning the new mineralized bone and the presence of graft material. Lévai-Laczkó staining is able to differenciate new and old mineralized bone (new bone intense violet-blue, old paler tone). Bone substitute is shown in grey. Magnification x40.

For each central section, the following variables were assessed (23) (Fig. 2):

1. Defect area diameter or former defect in mm (descriptive variable).

2. Target Area (TA): surface occupied by bone expressed as mm2 (descriptive variable).

3. Augmented Area (AA): mineralized bone matrix and non-mineralized tissue and/or bone substitute expressed as mm2 and percentage of the AA within the TA (primary outcome variable).

4. Mineralized Bone Matrix (MBM): according to the recommendations of the American Society for Bone and Mineral Research (ASBMR) to standardize bone histomorphometric nomenclature (24), was defined as the mineralized tissue within AA expressed as mm2 and percentage.

5. Bone Substitute (BS): remaining graft particles within AA expressed as mm2 and percentage.

6. Gained tissue (GT): total volume of MBM expressed as mm2 and percentage.

7. Graft Perimeter (GP): continuous line forming the boundary of the graft in TA expressed as mm.

8. Graft Surface to MBM (GS-MBM): the surface fraction of RBS in contact with newly formed MBM expressed as percentage.

- Statistical analysis

The specimens’ characteristics were calculated as absolute and relative frequencies for categorical outcomes. Normality of scale variables was explored using the Shapiro-Wilk test and through the visual analysis of the P-P plot and box plot. Where normality was rejected, the interquartile range (IQR) and median were calculated. Where distribution was compatible with normality, the mean and standard deviation (SD) were used.

To compare the treatments (β-TCP-*Fn*, β-TCP or control) and to analyze the effect of time (2 or 6 weeks) and the interaction between these two variables, a mixed model was used considering the animal as a random factor. All models were validated qualitatively exploring graphically the distribution of the residuals. For each follow-up time, pairwise comparisons between groups were performed.

The statistical analysis was carried out with the R software v3.1.2 (Development Core Team 2008). The level of significance was set at *P* < 0.05, using Tukey’s correction for multiplicity of contrasts.

## Results

All rats were treated without registering any deviation from the protocol. Therefore, 30 animals were studied.

Histomorphometric analysis was performed in 60 samples, 30 collected at 2 weeks and 30 at 6 weeks. There were 15 (25.0%) samples in the β-TCP-*Fn* group (8 collected at 2 weeks and 7 at 6 weeks), 15 (25.0%) in the β-TCP group (7 collected at 2 weeks and 8 at 6 weeks) and 30 (50%) in the untreated controls (15 collected at 2 weeks and 15 at 6 weeks).

The mean (SD) diameter of the bone defect was 4.55 [0.42] mm and comparable regarding treatment groups (*p* = 0.251) and weeks (*p* = 0.631). The mean target area was 4.35 [0.68] mm2, with significant differences (*p* = 0.019) between β-TCP and controls (4.59 [0.66] vs. 4.16 [0.64] mm2; *p* = 0.030; Table 1).

- AA

**Figure 3:**
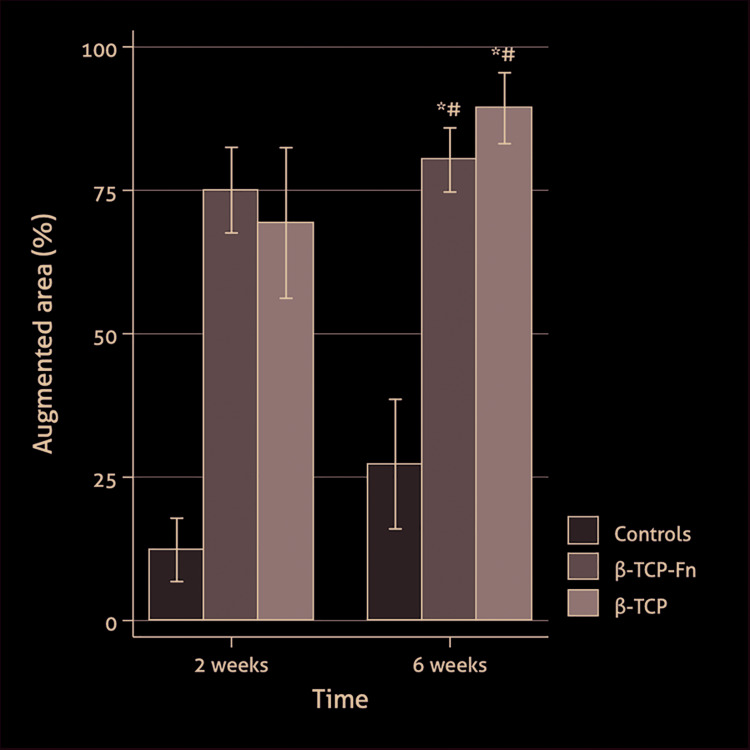
Percentage of augmented area (AA) within the target area by treatment groups and study periods (data expressed as mean and standard deviation); *#statistically significant differences for the comparison of β-TCP-*Fn* and β-TCP vs. controls.

Significant differences between the three study groups were found, with significantly higher mean values for the two treatment groups (β-TCP-*Fn* and β-TCP) when compared to controls (77.5% [7.9] and 80.0% [14.9], respectively, vs. 19.8% [17.5]; *p* < 0.001; Table 1). Also, at 2 and 6 weeks, the AA was significantly higher in the two grafted treatment groups than in the controls (Table 1, Fig. 3). An increase in AA was observed between 2 to 6 weeks, especially in the β-TCP (from 69.3% to 89.3%; *p* < 0.001; Table 1) and control groups (from 12.3% to 27.3%; *p* < 0.001; Table 1). It is important to note that after two weeks the defects filled with β-TCP-*Fn* have a higher AA than those filled in with β-TCP (75.03 [8.93] vs. 69.31 [14.20]) which is congruent with a faster increase in bone formation.

- MBM

In relation to the effect of the graft materials in the MBM, no significant differences were found in absolute (mm2) nor relative (%) terms between the treatment groups and the controls (Table 1). Although a significant increase in MBM was observed from 2 to 6 weeks in all the study groups, the magnitude of the change was greater in the active groups than in the controls (β-TCP-*Fn* from 0.21 [0.32] mm2 to 0.71 [0.51] mm2 and 1.4% [1.4] to 16.3% [9.8]; β-TCP from 0.06 [0.05] mm2 to 0.81 [0.55] mm2 and 2.5% [3.5] to 16.3% [11.11]; controls from 0.20 [0.18] mm2 to 0.62 [0.53] mm2 and 5.0% [4.4] to 15.3% [11.9]; Table 1). Again, MBM was greater at 2 weeks for β-TCP-*Fn* in mm2 and % but very similar at 6 weeks.

- Residual BS (RBS)

The percentage of bone substitute within the target area at 2 weeks in the two active treatment groups was significantly higher in the β-TCP-*Fn* (48.7% [5.7]) than in the β-TCP group (39.3% [7.7]; *p* = 0.040; Table 1). Similar results were obtained when BS was expressed as absolute area (mm2). There was a significant trend for a decrease in BS area in the β-TCP-*Fn* group from 2 to 6 weeks (2.24 [0.58] mm2 and 1.70 [0.41] mm2; *p* = 0.031).

- Gained tissue (GT)

Mean of GT expressed in percentage (MBM and residual BS) within the target area was significantly higher in the β-TCP-*Fn* group than in the controls (*p* = 0.028; Table 1). When GT was expressed as absolute area (mm2), the same statistical differences were obtained when comparing β-TCP-*Fn* group to controls was seen (*p* = 0.011; Table 1). At two weeks there is a tendency to a better result in terms of GT expressed in percentage for β-TCP-*Fn* vs. controls at two weeks (*p*=0.056).

**Table 1:**
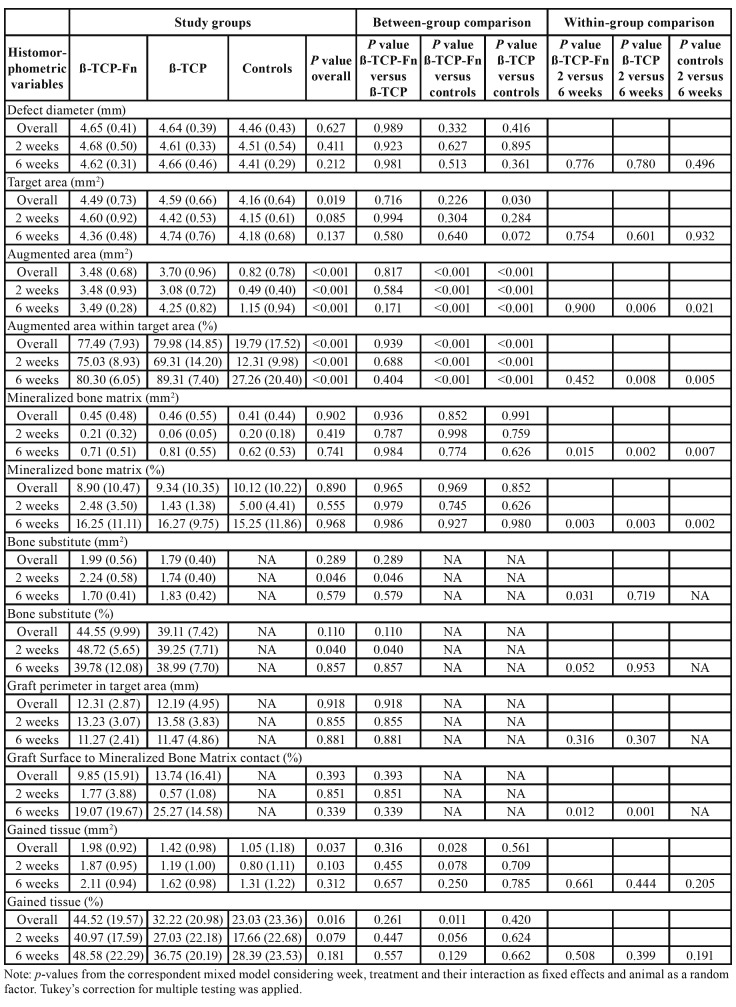
Results of histomorphometric variables in the three study groups. Data expressed as mean (SD); NA: not applicable.

This event is not observed for β-TCP alone. No differences between β-TCP-*Fn* and β-TCP were observed in terms of GT (*p* = 0.316; Table 1).

- Graft perimeter

The overall GP amounted to 12.31 [2.87] mm for β -TCP-*Fn* and 12.19 [4.95] mm for β-TCP, due to the similar amount of particles inserted into the defects. Although there were no statistically significant differences between the grafted defects with β-TCP-*Fn* or β-TCP, a slight reduction in GP was noticed in both of the active groups (Table 1).

- Graft Surface to MBM contact

When assessing the GS-MBM, the highest mean values were reached for the β-TCP group, but differences were not statistically significant (*p* > 0.05; Table 1). Within-group comparisons there is a significant increase in GS-MBM between 2 and 6 weeks in CSDs treated with β-TCP-*Fn* (from 1.77 [3.88] % to 19.07 [19.67] %; *p* = 0.012; Table 1) and β-TCP (from 0.57 [1.08] % to 25.27 [14.58] %; *p* = 0.001; Table 1).

## Discussion

This study shows that the addition of fibronectin to β-TCP grafted material was effective to improve bone regeneration in calvarial CSDs in a rat model respect to the negative controls in some histomorphometric parameters. GT, defined as MBM plus residual BS, measured in mm2 and as a percentage of the target area obtained the best result when compared β-TCP-*Fn* to the controls, specially at short time (two weeks of healing). Mean of GT within the target area was significantly higher in the β-TCP-*Fn* group than in the controls (*p*=0.028). When GT was expressed as absolute area (mm2), the same statistical differences were obtained when comparing β-TCP-*Fn* group to controls (*p*=0.011) and there is an almost statistically significant difference in favor related to the percentage at two weeks (*p*=0.056).

These results at that time point (2 weeks), could be a consequence of two factors: a greater amount of MBM in mm2 (2.24 [0.58]) and in percentage (2.48 [3.50]) and a significantly greater amount of BS in mm2 (2.24 [0.58]) and in percentage (48.72 [5.65]) in contrast with β-TCP alone for the same parameters ((0.06 [0.05], 1.43[1.38], 1.74 [0.40], 39.25 [7.71] respectively).

In return, looking MBM results at 6 weeks, it can be observed a change in the behavior of the alloplastic modified material having almost de same Figure for the two test groups both in mm2 (0.71 [0.51] for β-TCP-*Fn* vs. 0.81 [0.55]) and in percentage (16.25 [11.11] for β-TCP-*Fn* vs. 16.27 [9.75]), possibly as a consequence of a faster mineralization when *Fn* is added to β-TCP, and a greater ratio of resorption of the bone substitute throughout the experimentation period as Rojbani *et al*. observed using β-TCP coated with simvastatin (8). These results can be interesting in terms of clinical applicability.

Respect of the AA within the CSDs, the amount of bone regenerated was similar in the study groups independently of the time period (*p*=0.817), but -*Fn* group maintain the same volume in mm2 along the time (3.48 [0.93] at 2 weeks, 3.49 [0.28] at 4 weeks) and conversely β-TCP group reached this volume progressively increasing. β-TCP-*Fn* seems more effective respect to the volume maintenance since the grafting time. In a previous study, the same variables where evaluated but with longer healing time (6 and 8 weeks). At 8 weeks MBM in mm2 was observed a significant difference in favor of β-TPC-*Fn* respect to the β-TPC alone (*P*=0.04). Changes of bone regeneration according to materials used and time of analysis also suggest a more favorable effect of β-TCP-*Fn* as the percentage of MBM in the target area was further increased from week 6 to week 8 (*P*=0.067) (21).

Only Calciolari *et al*. (25) studying collagen membranes degradation for bone regeneration purposes in calvarial CSDs, used shortest time periods to evaluate the thickness and histological events of the membrane but not the quality or quantity of the regenerated bone (7, 14 and 30 days) as our experimental model. Ramalingam *et al*. (26) compare guide bone regeneration using β-TPC and collagen membrane in an *in vivo* model with micro-CT of 3.3. diameter calvarial defects at 2, 4, 6 and 10 weeks but doesn’t retrieve histological data until the 10th week, when finally sacrificed the rats. Kostopoulos & Karring (27) analyzed osseous regeneration in 2x3 mandibular bone defects covered with a polyhidroxybutyrate acid membrane at 7, 15, 30, 90 and 180 days in terms of histologic composition and percentage of filing of the original defect specifying that at 1-month controls were completely repaired. Then, few possibilities of comparison could be done at 2 weeks period amongst de effects of other filling materials for bone regeneration and β-TCP-*Fn*. Most of the authors evaluates the samples between 4, 6, 8, 10 weeks (28-31). This short time experimental period of two weeks also guarantees that the spontaneous healing of the defect does not occur, as it was in some cases, using equal size defects as in a previous study (21).

Microporosity, crystallinity and size of the β-TCP particle seem crucial to provide an optimal structure for vascular growth and bone formation. Microporosity (pore size) < 10 µm increases macromolecule adhesion and favor fluid penetration, although a highly porous β-TCP material (> 100 µm) also supported new bone formation (32). On the other hand, reducing the size of β-TCP granules to nanometers may also contribute to induce higher porosity and larger specific surfaces, leading to an improved regenerative effect (7). In our study, the pore size of KeraOs® was between 100-250 µm, but future studies should be focused on assessment of the performance of β-TCP materials with smaller particle size.

Fibronectin has been used to stimulate mineralization and cell adhesion in tricalcium phosphate scaffolds, resulting in early differentiation of osteoblasts (16). In a novel multilayered chitosan-hydroxyapatite composite, the addition of *Fn* (25 or 50 µg/ml) improved osteoblast cell adhesion and proliferation, demonstrating the potential of fibronectin for improving the quality of this material as a bone graft (17). In our study the concentration of fibronectin is coated to a 1 gr of a β-TCP scaffold but 10 µg/ml. Comparisons about concentrations cannot be calculated because the data in the study cited (17) are expressed in mm3 for the scaffold and in 10 µl/ml for fibronectin.

Is suggested in an *in vitro* study using a *Fn*-derived oligopeptide, that fibronectin also induces osteoblast differentiation mediated by BMP-2 and can be used as a therapeutic biomolecule to facilitate even periodontal regeneration (33). Other attempts to improve β-TCP scaffolds has been done with simvastatin that seems to stimulate BMP-2 expression of osteoblasts, combined with three different calcium phosphate biomaterials (α-TCP, β-TCP and hydroxyapatite) in a rat calvarial defect model (8). The results showed that simvastatin also affected the α-TCP and β-TCP degradation, and especially when combined to α-TCP, which showed a higher degradation rate allowing more bone formation (8,34).

Other associations to β-TCP are: growth factors (11,35) dental pulp stem cells (14,15), with inconsistent results, adipose stem cells (19,20), BMP-2 that did not substantially changed the osteoconductive properties of the biomaterials grafted as compared with TCP alone (12), BMP-2 in 3D printed polycaprolactone/ β-TCP plus decellularized extracellular matrix with significant improvement (*p*<0.01) when compared with controls (29).

In the present study, calvarial defects in controls gained less volume than those in the remaining groups because grafts helped to maintain the original bonny space confirming previous research (21), being the AA in percentage of the target area 75.03 [8.93] for β-TCP-*Fn*, 69.31 [14.20] for β-TCP and 12.31 [9.98] for controls at two weeks (*p*=0.001), taking into account that also controls were covered with collagen membrane and, as it has been demonstrated, this treatment is a benefit for bone regeneration at an early stage giving enough time to the bone cells to refill the hard tissue defect (36).

Barrier membrane in our study did not influence to the ability to be an effective negative control allowing to obtain statistically significant results between the test and control groups at different times. Donos *et al*. (37) with a similar experimental model, concluded that the use of a barrier membrane alone has the same efficacy as the use of regeneration materials, nevertheless, the time of euthanasia of the experimental model was 16 weeks, so it could be thought that CSDs 5 mm in diameter was too little for their study time, although it can be considered adequate in rat model for other periods. Even so, the authors conclude that the control’s healing occurred with a significant regeneration deficit in height (38).

Findings of the study should be interpreted taking into account some limitations. A potential source of bias related to the differences between individuals done by the size and thickness of the calvaria. This drawback was corrected by expression the results of histomorphometric variables as percentages of the target area.

Regarding the bilateral 5 mm diameter for the CSDs, it seems sufficient to obtain relevant data (38). Although the design used allows reduce the risk of bias by having the tested material and the control in the same animal, it can be a risk in terms of contamination of the control defect (38) in our case, no one contaminated control sample was found in any group and any time.

Although no species fulfils the requirements of an ideal animal model, rodents are one of the best choices because they are easily available, easy to house and handle. Also, a large number of studies on regeneration of bone defects published in the literature have been carried out in rat models. In addition, the rat calvarial bone model allows establishing a standardized and reproducible defect.

The applicability of this findings could be interesting due to the maintenance of the bone volume pretended to regenerate and the faster mineralization of the tissue gained leading a safe and more predicTable results when an osseous regeneration is necessary.

## Conclusions

With the limitations of this study, β-TCP with and without fibronectin supposed an advantage in maintaining the volume regenerated, avoidance of soft tissue invading the hard tissue space and acceleration of the new bone formation process when compared with bone defects left empty.

Gained tissue in defects filled with β-TCP coated with fibronectin is significantly better when compared with controls this event is not confirmed for β-TCP used alone in rats calvarial CSDs.
